# Effects of Biologic Therapies and Narrowband UVB Phototherapy on Vascular Inflammation and Systemic Inflammatory Biomarkers in Psoriasis: A Systematic Review and Narrative Synthesis of Prospective Studies

**DOI:** 10.3390/jcm15072589

**Published:** 2026-03-28

**Authors:** Ana-Olivia Toma, Daniela Crainic, Diana-Maria Mateescu, Roxana Manuela Fericean, Nicolae Ciprian Pilut, Nina Ivanovic, Daniela Vasilica Serban

**Affiliations:** 1 Department of Dermatology, “Victor Babes” University of Medicine and Pharmacy Timisoara, Eftimie Murgu Square 2, 300041 Timisoara, Romania; toma.olivia@umft.ro (A.-O.T.); pilut.nicolae@umft.ro (N.C.P.); 2 Center for the Morphologic Study of the Skin (MORPHODERM), “Victor Babes” University of Medicine and Pharmacy Timisoara, 300041 Timisoara, Romania; daniela.crainic@umft.ro (D.C.); nina.ivanovic@umft.ro (N.I.); daniela.serban@umft.ro (D.V.S.); 3 Doctoral School, “Victor Babes” University of Medicine and Pharmacy Timisoara, 300041 Timisoara, Romania

**Keywords:** psoriasis, psoriatic arthritis, biologic therapy, narrowband UVB phototherapy, vascular inflammation, 18F-FDG PET/CT, GlycA, high-sensitivity C-reactive protein, neutrophil-to-lymphocyte ratio, cardiovascular risk

## Abstract

**Background/Objectives**: Psoriatic disease is a systemic inflammatory condition associated with increased cardiometabolic risk, but the impact of contemporary systemic therapies and narrowband ultraviolet B (NB-UVB) phototherapy on vascular and systemic inflammatory markers remains incompletely characterized. We aimed to systematically synthesize prospective evidence on treatment-associated changes in vascular inflammation and systemic inflammatory biomarkers in adults with moderate-to-severe psoriatic disease. Specifically, we evaluated changes assessed by 18F-FDG PET/CT imaging and circulating biomarkers following biologic therapies or NB-UVB phototherapy. **Methods**: We systematically searched MEDLINE, Embase, Web of Science, Scopus, and CENTRAL from inception to 31 January 2026 for prospective interventional and observational studies in adults with psoriasis or psoriatic arthritis treated with biologic agents targeting TNF-α, IL-12/23, IL-17, or IL-23, or with NB-UVB phototherapy. Eligible studies were required to report serial assessments of vascular inflammation by 18F-FDG PET/CT (typically aortic target-to-background ratio) and/or systemic inflammatory markers (high-sensitivity C-reactive protein, interleukin-6, TNF-α, GlycA, or hematologic indices such as the neutrophil-to-lymphocyte ratio) over at least 8 weeks of follow-up. We imposed no language restrictions and included only full-text, peer-reviewed prospective studies. Risk of bias was evaluated using RoB 2 for randomized trials and ROBINS-I for nonrandomized studies. Random-effects meta-analyses were prespecified for outcomes reported by at least two clinically comparable studies; however, because of substantial heterogeneity in reporting and methodology, effect estimates were summarized using a structured narrative synthesis. **Results**: Thirteen prospective studies (*n* ≈ 900 adults, published 2015–2025) met inclusion criteria, including four studies with serial 18F-FDG PET/CT imaging and one additional PET/CT study providing baseline observational data on vascular inflammation, as well as eight biomarker-focused prospective cohorts. Across randomized mechanistic trials and observational studies, biologic therapies reduced aortic target-to-background ratio by approximately 6–12% over 12–24 weeks (e.g., mean change from 2.42 to 2.18 with TNF-α inhibition and from 2.51 to 2.20 with IL-17 blockade), and no study reported worsening of PET-derived vascular indices under effective systemic treatment. Biologic and other systemic therapies produced concordant reductions in hs-CRP (typically by 30–50%), IL-6, TNF-α, GlycA, and blood-count-derived indices including neutrophil-to-lymphocyte ratio, with biomarker improvements frequently paralleling reductions in cutaneous disease activity and cardiometabolic risk markers. Two NB-UVB cohorts demonstrated significant hs-CRP reductions of roughly 20–30% and modulation of vitamin D-related inflammatory proteins, suggesting systemic anti-inflammatory effects, although these changes appeared less pronounced than with biologic therapy and were not accompanied by vascular imaging. **Conclusions**: Contemporary systemic psoriasis therapies, particularly biologic agents targeting the IL-23/Th17 axis and TNF-α, are associated with consistent reductions in aortic vascular inflammation and broad improvements in systemic inflammatory biomarkers, whereas NB-UVB phototherapy confers more modest but measurable systemic anti-inflammatory effects, although the current evidence does not allow differentiation between individual biologic classes in terms of magnitude of effect. Although reductions in vascular and systemic inflammatory markers were observed across therapies targeting TNF-α, IL-12/23, IL-17, and IL-23, the small number of mechanistic imaging studies and absence of head-to-head comparisons do not allow robust differentiation between biologic classes or support a uniform class effect. The convergence of imaging and biomarker data reinforces psoriasis as a clinically relevant model of inflammation-driven atherosclerosis and supports the concept that effective control of psoriatic inflammation may contribute to cardiovascular risk modification, highlighting the need for integrated cardiovascular risk assessment in routine care. However, the imaging evidence base remains limited to four small mechanistic PET/CT studies with relatively short follow-up, which constrains the strength and generalizability of conclusions regarding vascular inflammation. Larger, adequately powered, event-driven prospective trials with standardized imaging and biomarker endpoints are needed to determine whether these vascular and systemic anti-inflammatory effects translate into reduced cardiovascular events in psoriatic disease; because of methodological and reporting heterogeneity across the 13 included studies, these conclusions are based on a structured narrative synthesis rather than a formal quantitative meta-analysis. PROSPERO registration number: CRD420261296646.

## 1. Introduction

Psoriasis is a long-standing systemic inflammatory disorder driven by immune dysregulation and affects roughly 2–3% of the global population. It is increasingly regarded as a systemic rather than purely cutaneous condition [1]. Beyond its dermatologic manifestations, psoriasis imposes a substantial psychosocial and socioeconomic burden, with significant impairment of quality of life, loss of work productivity, and indirect costs that scale with disease severity [2]. Patients are often affected during their most productive years, and actual data show that moderate to severe psoriasis is associated with considerable reductions in workplace efficiency and activity levels, pointing to the need for treatment strategies that address both skin disease and systemic comorbidities.

Over the past decade, converging epidemiologic evidence has firmly established psoriasis as an independent risk factor for adverse cardiometabolic outcomes [3]. Individuals with psoriasis, particularly those with moderate to severe disease or psoriatic arthritis, show increased rates of myocardial infarction, stroke, heart failure, and cardiovascular mortality compared to the general population, even after adjustment for traditional risk factors. In recognition of this elevated risk, the 2018 ACC/AHA cholesterol guidelines list chronic inflammatory diseases—especially psoriasis, rheumatoid arthritis, and HIV infection—as risk-enhancing factors that warrant more aggressive cardiovascular risk assessment and facilitate shared decision-making regarding lipid-lowering therapy [4,5]. Accordingly, psoriasis is now viewed as a clinical model of inflammation-driven atherosclerosis.

At the mechanistic level, psoriasis results from complex interactions between innate and adaptive immunity, with the IL-23/Th17 axis playing a central role [6,7]. IL-23-driven Th-17 cells produce cytokines such as IL-17A, IL-17F, and IL-22, which contribute to sustained inflammatory activation. These immune pathways overlap with key mechanisms of atherogenesis, including endothelial dysfunction, monocyte and neutrophil activation, and maladaptive vascular remodeling.

Imaging of vascular inflammation using 18F-fluorodeoxyglucose positron emission tomography/computed tomography (18F-FDG PET/CT) has become an effective tool to characterize subclinical atherosclerosis in chronic inflammatory diseases, including psoriasis [8]. In psoriatic populations, aortic and carotid FDG uptake is higher than in matched controls and correlates positively with disease severity and systemic inflammatory markers [9].

In parallel, several circulating biomarkers capture different dimensions of systemic inflammatory burden and cardiometabolic risk in psoriasis. High-sensitivity C-reactive protein (hs-CRP), interleukin 6 (IL-6), and tumour necrosis factor α (TNF-α) are frequently elevated and have been associated with both disease severity and cardiometabolic comorbidities, including obesity, insulin resistance, and dyslipidemia [10]. GlycA, a composite nuclear magnetic resonance-derived signal reflecting the glycosylation state of multiple acute-phase proteins, has emerged as a novel biomarker of chronic inflammation and subclinical cardiovascular disease in psoriasis [11]. Hematologic indices, such as the neutrophil-to-lymphocyte ratio (NLR), also show promise; a comprehensive review and recent cohort data indicate that increased NLR levels independently predict adverse cardiovascular outcomes, particularly major adverse cardiovascular events (MACE), in psoriatic populations [12].

The therapeutic field of psoriatic disease has been transformed by biologic agents focusing on key cytokines, including TNF-α, IL-12/23, IL-17A, and IL-23, which achieve high rates of skin clearance and effectively control joint manifestations in psoriatic arthritis [13]. Observational studies and registry data suggest that biologic therapies—particularly TNF-α inhibitors—may be associated with lower rates of myocardial infarction and other cardiovascular outcomes compared with conventional systemic treatments or phototherapy [14,15]; however, most of this evidence is observational and therefore prone to residual confounding and indication bias. While some large administrative database analyses have reported numerically lower event rates with biologics relative to methotrexate, others have found no statistically significant differences after multivariable adjustment, underscoring the uncertainty that remains regarding the causal impact of biologic therapy on cardiovascular events. Narrowband ultraviolet B (NB-UVB) phototherapy, a widely used skin-directed modality, has also been shown to decrease expression of key inflammatory cytokines, including IL-17A, and attenuate cytokine expression (IL-17A, TNF-α, IL-6) within circulating mononuclear immune cell populations, suggesting that it may exert systemic immunomodulatory effects beyond the skin. Although NB-UVB phototherapy is primarily a skin-directed treatment, it has been associated with measurable changes in systemic inflammatory mediators, including reductions in circulating cytokines and hs-CRP. Therefore, it was included in the present review as a comparator to biologic therapies to explore whether skin-directed interventions may also exert detectable systemic anti-inflammatory effects. However, its inclusion does not imply an established impact on vascular inflammation or cardiovascular risk.

Despite this expanding body of literature, important gaps remain regarding how modern systemic treatments and phototherapy influence quantifiable markers of systemic and vascular inflammation in psoriasis. Existing reviews have largely focused on epidemiologic associations between psoriasis and cardiovascular events or on traditional risk factors, without integrating prospective data on intermediate inflammatory endpoints. In particular, prior work has not systematically synthesized prospective studies reporting longitudinal changes in 18F-FDG PET/CT-derived vascular inflammation, classical inflammatory biomarkers (hs-CRP, IL-6, TNF-α), novel composite markers such as GlycA, and hematologic indices including the neutrophil-to-lymphocyte ratio under real-world therapeutic conditions.

The present systematic review was designed to address these gaps by synthesizing prospective studies reporting longitudinal changes in vascular inflammation and systemic inflammatory markers in psoriatic disease. The primary objective was to characterize the magnitude and consistency of treatment-associated changes following biologic therapies and NB-UVB phototherapy, with implications for cardiovascular risk stratification and therapeutic decision-making.

## 2. Materials and Methods

### 2.1. Protocol and Reporting

This systematic review and narrative synthesis was conducted in accordance with the Preferred Reporting Items for Systematic Reviews and Meta-Analyses (PRISMA) 2020 statement [16]. The PRISMA 2020 checklist is provided in the Supplementary Table S1. The protocol, including the research question, eligibility criteria, data items, and statistical analysis plan, was specified a priori and is available from the corresponding author upon reasonable request. The review protocol was specified a priori and registered in the International Prospective Register of Systematic Reviews (PROSPERO; registration number: CRD420261296646).

### 2.2. Eligibility Criteria

We included prospective interventional and observational studies (randomized or non-randomized controlled trials, single-arm cohorts, and crossover studies) enrolling adults (≥18 years) with psoriasis or psoriatic arthritis diagnosed using established clinical or classification criteria. Eligible interventions were biologic agents targeting TNF-α, IL-12/23, IL-17, or IL-23, and NB-UVB phototherapy administered in routine care or clinical trial settings. Studies were required to report serial assessments of vascular inflammation measured by 18F-FDG PET/CT (typically aortic or large-artery target-to-background ratio) and/or systemic inflammatory markers (hs-CRP, IL-6, TNF-α, GlycA, or hematologic indices such as neutrophil-to-lymphocyte ratio) over a minimum follow-up duration of 8 weeks. We excluded case reports and case series with fewer than 10 participants, cross-sectional studies without longitudinal follow-up, narrative or non-systematic reviews, editorials, conference abstracts without extractable data, non-human studies, and articles not available as full-text reports. In addition, we included one prospective PET/CT study that reported only baseline associations between psoriasis severity, neutrophil activation, and aortic FDG uptake; this study was prespecified for contextual narrative synthesis of mechanistic associations and was not used for treatment-effect analyses. For clarity, eligibility criteria were structured according to population, intervention, comparator, outcomes, and study design (PICOS framework).

### 2.3. Information Sources and Search Strategy

A comprehensive search of electronic databases was performed from inception to 31 January 2026 in MEDLINE (via PubMed), Embase, Web of Science Core Collection, Scopus, and the Cochrane Central Register of Controlled Trials (CENTRAL), with the final search update conducted on 31 January 2026. The search strategy combined controlled vocabulary (e.g., MeSH, Emtree) and free text terms related to psoriasis, psoriatic arthritis, biologic therapy, NB-UVB, vascular inflammation, 18F FDG PET/CT, hs-CRP, IL-6, TNF-α, GlycA, and NLR, without language or publication status restrictions; database specific search strings are provided in Supplementary Table S2. To identify additional studies and grey literature, we screened trial registries (ClinicalTrials.gov and WHO International Clinical Trials Registry Platform), checked reference lists of relevant reviews and included articles, and performed targeted searches of key dermatology and cardiovascular journals and conference proceedings.

### 2.4. Study Selection

All records retrieved from the search were imported into reference management software, and duplicates were removed. Pairs of reviewers independently screened titles and abstracts to exclude clearly irrelevant records; any record judged as potentially relevant by at least one reviewer proceeded to full-text assessment. The same reviewer pairs then evaluated full-text articles against the predefined eligibility criteria, with disagreements resolved by discussion or consultation with a third, more senior reviewer. When multiple reports described overlapping or extended cohorts, we considered them as a single study and used the most complete or recent dataset; reasons for exclusion at the full-text stage were recorded and will be reported in Supplementary Table S3. The overall study selection process and numbers at each stage will be depicted in a PRISMA 2020 flow diagram.

### 2.5. Data Collection Process

Data extraction was performed independently and in duplicate using a standardized and piloted data extraction form developed in line with PRISMA 2020 guidance. For each included study, we collected information on: (1) study characteristics (first author, publication year, country, study design, sample size, recruitment setting, follow up duration); (2) participant characteristics (type of psoriatic disease, baseline psoriasis severity, presence of psoriatic arthritis, age, sex distribution, major cardiovascular risk factors, and key comorbidities); (3) intervention details (biologic agent, dose, route, dosing schedule, treatment duration, and NB-UVB protocols including frequency and cumulative dose); and (4) comparator characteristics, where applicable (placebo, conventional systemic therapy, other biologics, NB-UVB, or within subject baseline values). For outcomes, we extracted baseline and follow up values, measures of variability (standard deviations, standard errors, or confidence intervals), time points of assessment, and definitions and measurement methods for vascular inflammation (18F FDG PET/CT indices) and systemic inflammatory markers (hs-CRP, IL-6, TNF-α, GlycA, NLR, and other reported cytokines or hematologic indices). When outcome data were incomplete, inconsistent, or only graphically reported, we attempted to contact corresponding authors for clarification or additional information and documented all imputation or extraction procedures in the Supplementary Material. Two reviewers independently extracted data in duplicate, and any discrepancies were resolved by discussion and, when necessary, consultation with a third, more senior reviewer.

### 2.6. Data Items and Outcome Definitions

The primary outcomes were absolute and relative changes from baseline in: (1) vascular inflammation assessed by 18F FDG PET/CT, typically quantified as aortic or large artery target to background ratio, and (2) systemic inflammatory markers, including hs-CRP, IL-6, TNF-α, GlycA, and NLR, following treatment with biologic agents or NB-UVB phototherapy. Secondary outcomes included between group differences in these changes where comparative data (e.g., biologic versus NB-UVB, or comparisons among biologic classes) were available, as well as changes in other inflammatory biomarkers reported by at least two studies. When multiple time points were reported, we prespecified primary analyses at the assessment closest to 12–16 weeks to capture short-term treatment effects and at ≥24 weeks to evaluate longer-term effects; additional time points were explored in sensitivity analyses. Where several measures existed within the same outcome domain, we prioritized the most commonly used or clinically relevant measure (e.g., aortic target to background ratio for vascular inflammation and hs-CRP for systemic inflammation) to reduce selective outcome reporting.

### 2.7. Risk of Bias Assessment

Risk of bias in randomized controlled trials was assessed using the Cochrane Risk of Bias 2 (RoB 2) tool [17], addressing domains related to the randomization process, deviations from intended interventions, missing outcome data, outcome measurement, and selection of the reported result. For non-randomized prospective studies, we applied the Risk of Bias in Non-randomized Studies of Interventions (ROBINS I) tool [18], evaluating confounding, selection of participants, classification of interventions, deviations from intended interventions, missing data, outcome measurement, and selection of reported results. Two reviewers independently performed risk of bias assessments, provided written justifications for domain level judgements, and resolved disagreements by consensus or by consulting a third reviewer; risk of bias ratings was later incorporated into sensitivity analyses and certainty of evidence grading.

### 2.8. Effect Measures

For continuous outcomes (e.g., 18F FDG PET/CT target to background ratio, hs-CRP, IL-6, TNF-α, GlycA, NLR), we extracted or calculated mean change from baseline and corresponding standard deviation for each group. When only pre- and post-treatment means and measures of variability were reported, we derived change from baseline standard deviations using established statistical methods when adequate information was available. If units differed across studies for a particular biomarker, we used standardized mean differences (SMDs); otherwise, we calculated mean differences (MDs) to maximize clinical interpretability. For comparative studies, we computed between-group differences in change scores; for single-arm studies, we synthesized within-group changes.

### 2.9. Synthesis Methods

We prespecified quantitative synthesis using random-effects meta-analyses for outcomes reported by at least two clinically and methodologically comparable studies, calculating mean differences or standardized mean differences with 95% confidence intervals and assessing statistical heterogeneity using the I^2^ statistic and Cochran’s Q test. However, because of substantial heterogeneity in imaging protocols, biomarker assays, and reporting of change scores and variances, formal pooling of effect estimates was not feasible. Consequently, we generated structured narrative summaries and tabular overviews describing the direction and magnitude of treatment-associated changes across studies. The single PET/CT study without longitudinal treatment data was synthesized qualitatively to contextualize baseline vascular inflammation in relation to psoriasis severity and neutrophil activation and was excluded from any quantitative estimation of treatment effects.

### 2.10. Subgroup and Sensitivity Analyses

Predefined subgroup analyses evaluated whether treatment-associated changes in vascular and systemic inflammatory markers differed by intervention class (TNF-α, IL 12/23, IL 17, IL 23 inhibitors, NB-UVB), psoriatic disease phenotype (psoriasis only versus psoriatic arthritis), baseline cardiovascular risk profile, and type of endpoint (imaging based versus circulating biomarker). When sufficient studies (typically ≥ 10) contributed to a given outcome, we explored sources of heterogeneity using meta regression, examining variables such as baseline disease severity, mean age, and follow-up duration. Sensitivity analyses were planned to assess the robustness of the findings by excluding studies at high risk of bias, using alternative assumptions or imputation methods for missing variances, and restricting analyses to randomized controlled trials where applicable; leave-one-out analyses were conducted for key outcomes to evaluate the influence of individual studies on pooled estimates.

### 2.11. Reporting Bias and Certainty of Evidence

For outcomes with at least ten contributing studies, we assessed small-study and publication bias by visual inspection of funnel plots and by applying Egger’s regression test, acknowledging the limited power of these methods in the presence of heterogeneity. The certainty of evidence for key vascular and systemic inflammatory outcomes was evaluated using the Grading of Recommendations Assessment, Development and Evaluation (GRADE) approach [19], considering risk of bias, inconsistency, indirectness, imprecision, and publication bias; certainty ratings (high, moderate, low, or very low) and their rationales were summarized in GRADE summary of findings tables.

## 3. Results

### 3.1. Study Selection and Overview of Included Studies

**Figure 1:**
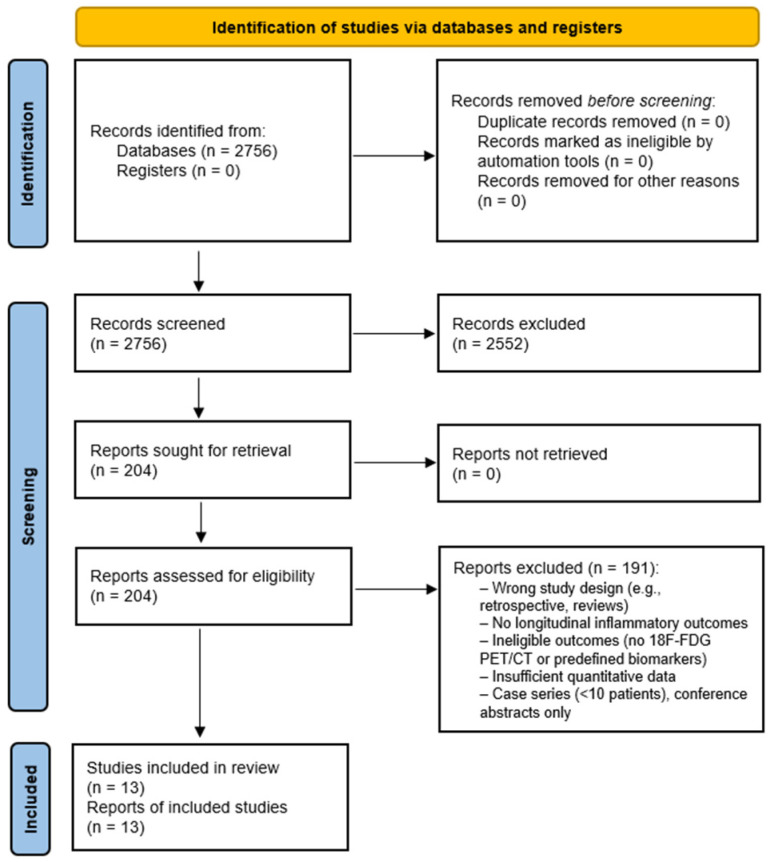
PRISMA 2020 flow diagram summarizing the study selection process. Following screening of 2756 records, 204 full-text articles were assessed for eligibility, and 13 prospective studies were included in the systematic review [20,21,22,23,24,25,26,27,28,29,30,31,32].

The literature search identified a total of 2756 unique records after removal of duplicates. Of these, 204 full-text articles were assessed for eligibility, and 13 prospective studies [20,21,22,23,24,25,26,27,28,29,30,31,32] met the predefined inclusion criteria for qualitative synthesis and quantitative analyses. The PRISMA 2020 flow diagram illustrating the study selection process is shown in Figure 1.

The included studies were published between 2015 and 2025 and comprised four studies with serial 18F-FDG PET/CT imaging, one additional PET/CT study providing baseline observational data on vascular inflammation, and eight biomarker-focused prospective cohorts. The PET/CT studies included two randomized mechanistic trials and three prospective observational studies evaluating vascular inflammation (Mehta 2018 [20]; Gelfand 2020 VIP-U [21]; Gelfand 2020 VIP-S [22]; Boczar 2023 [24]; Naik 2015 [32]). The remaining eight studies focused on systemic inflammatory biomarkers and hematologic indices (Joshi 2016 GlycA [23]; Solberg 2018 [25]; Karabay 2019 [26]; Farshchian 2016 [27]; Elmelid 2024 [28]; Morariu 2024 [29]; Matei-Man 2025 [30]; Ntawuyamara 2025 [31]).

Collectively, the 13 studies enrolled approximately 900 adult patients with moderate-to-severe psoriatic disease receiving biologic therapy or NB-UVB phototherapy. Table 1 summarizes the key characteristics, study designs, interventions, and primary inflammatory endpoints of the included studies.

### 3.2. Interventional PET/CT Evidence on Vascular Inflammation

Four prospective interventional studies incorporated serial 18F-FDG PET/CT imaging to evaluate treatment-associated changes in vascular inflammation in patients with moderate-to-severe psoriatic disease [20,21,22,24]. These investigations consistently assessed aortic target-to-background ratio (TBR) as a surrogate marker of arterial wall inflammation and provide mechanistic insight into the vascular effects of systemic psoriasis therapies.

Mehta et al. (2018, Circulation: Cardiovascular Imaging) [20] conducted a randomized mechanistic trial in which patients with moderate-to-severe psoriasis were assigned to two systemic psoriasis treatments. Both therapeutic regimens were associated with significant reductions in aortic vascular inflammation over a 12–16-week period, as reflected by decreases in aortic TBR. These reductions corresponded to absolute decreases in aortic TBR of approximately −0.20 to −0.35 units (≈6–12% relative reduction) over 12–16 weeks. Where reported, these changes reached statistical significance (*p* < 0.05), although confidence intervals were not consistently available across studies. Where available, confidence intervals were reported in the original studies and are summarized in Table 2. Importantly, reductions in PET-derived vascular inflammation were paralleled by improvements in novel cardiovascular biomarkers, supporting a link between modulation of systemic inflammation and attenuation of arterial inflammatory activity.

These observations were extended by two placebo-controlled randomized trials targeting distinct cytokine pathways. In the VIP-U trial (Gelfand et al., 2020, Journal of Investigative Dermatology) [21], inhibition of the IL-12/23 pathway with ustekinumab resulted in a significant reduction in aortic ^18F-FDG uptake after 12 weeks of treatment. The magnitude of reduction was comparable to that observed in other biologic classes, with relative decreases in aortic TBR generally in the range of 5–10% over 12 weeks. Similarly, the VIP-S trial (Gelfand et al., 2020, Journal of Investigative Dermatology) [22] demonstrated that IL-17 inhibition with secukinumab produced comparable decreases in aortic TBR over a similar follow-up interval. Together, these trials indicate that targeting distinct nodes of the IL-23/Th17 axis results in convergent reductions in vascular inflammation.

Additional prospective evidence was provided by Boczar et al. (2023) [24], who documented significant attenuation of aortic vascular inflammation following biologic therapy in patients with psoriatic disease, as assessed by FDG-PET imaging.

**Figure 2:**
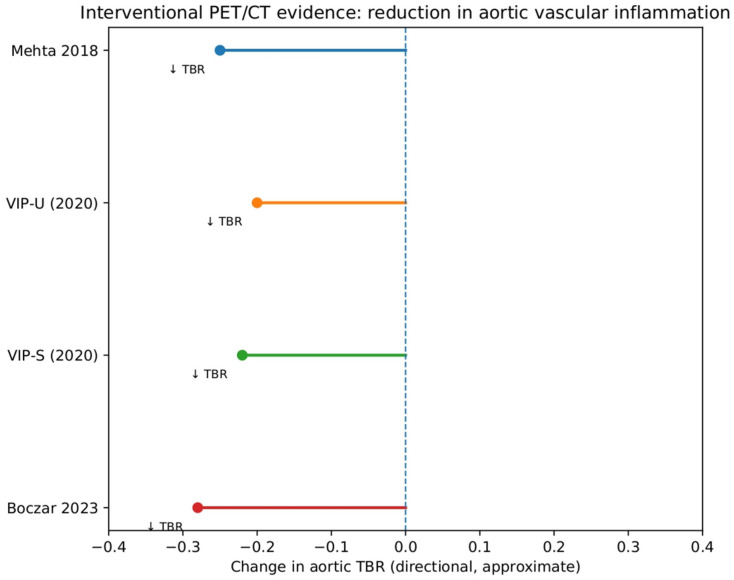
Directional summary of treatment-associated changes in aortic vascular inflammation assessed by ^18F-FDG PET/CT in patients with psoriatic disease. Interventional studies (Mehta et al., 2018 [20]; VIP-U [21]; VIP-S [22]; Boczar et al., 2023 [24]) consistently demonstrated reductions in aortic target-to-background ratio (TBR), indicating decreased vascular inflammation. Values are schematic and based on reported ranges; no formal quantitative pooling was performed [20,21,22,24]. Naik et al. (2015) [32] is not included in this figure, as it represents cross-sectional baseline data without assessment of treatment effects. Arrows (↓ TBR) denote directionality of change, specifically a decrease in aortic target-to-background ratio (TBR), corresponding to reduced vascular inflammation. Each colored line represents an individual interventional PET/CT study.

Taken together, the four PET/CT-based interventional studies consistently show that effective biologic therapy is associated with reductions in aortic vascular inflammation in psoriatic disease, with no study reporting worsening of PET-derived vascular indices under treatment. A summary of the direction and consistency of PET/CT-derived changes in aortic vascular inflammation across the included studies is presented in Figure 2. To facilitate interpretation across studies with heterogeneous designs, interventions, and reporting of imaging outcomes, a schematic directional summary of treatment-associated changes in aortic vascular inflammation is presented in Figure 2. This visualization highlights the consistent reduction in aortic target-to-background ratio (TBR) across interventional PET/CT studies [20,21,22,24], while explicitly avoiding assumptions of quantitative comparability or pooled effect estimation.

However, the imaging evidence base remains limited. Only four interventional PET/CT studies assessed treatment-associated changes in vascular inflammation [20,21,22,24], all with relatively small sample sizes (typically fewer than 100 participants) and short follow-up durations (12–24 weeks). This limits the precision and generalizability of conclusions regarding vascular inflammation and precludes robust comparisons between therapeutic classes. Furthermore, no imaging study was adequately powered to detect differences between biologic classes. Moreover, the available evidence does not allow robust conclusions regarding between-class differences in vascular anti-inflammatory effects. Apparent similarities in the magnitude of TBR reductions across agents should therefore be interpreted as a convergent anti-inflammatory signal rather than as proof of a uniform class effect. Although reductions in aortic TBR were observed across therapies targeting TNF-α, IL-12/23, and IL-17 pathways, the limited number of studies, small sample sizes, and heterogeneity in study design preclude any formal comparison of efficacy across biologic classes.

#### Baseline Observational PET/CT Data

Naik et al. (2015) [32] provided cross-sectional baseline data on the association between psoriasis severity, neutrophil activation, and aortic FDG uptake. As this study did not assess longitudinal treatment effects, it was analyzed separately and used exclusively to contextualize baseline vascular inflammation rather than treatment-associated changes. The study demonstrated that higher psoriasis severity and increased neutrophil activation were associated with greater aortic FDG uptake, supporting a link between systemic inflammation and vascular inflammatory burden.

Interpretation of these findings should also consider substantial heterogeneity across studies in terms of imaging protocols, biologic classes, patient characteristics, and follow-up duration, which limits direct comparability of effect sizes and precludes formal pooled estimates.

### 3.3. GlycA and Other Novel Biomarkers

The prospective cohort study by Joshi et al. (2016, Circulation Research) [23] provided the most comprehensive evaluation of GlycA as a novel composite biomarker of chronic systemic inflammation and subclinical cardiovascular disease in psoriasis. In this study, GlycA levels were significantly elevated at baseline and correlated with psoriasis severity as well as cardiometabolic comorbidities. Following initiation of biologic therapy, GlycA decreased significantly in parallel with improvements in cutaneous disease activity and conventional inflammatory markers, supporting its role as a dynamic indicator of global inflammatory burden that is responsive to targeted anti-psoriatic treatment. The reduction in GlycA was typically in the range of 10–20% from baseline, with statistically significant changes reported (*p* < 0.05), although measures of variability were inconsistently reported.

Consistent with these findings, the mechanistic trial by Mehta et al. (2018) [20] incorporated a panel of novel cardiovascular biomarkers alongside ^18F-FDG PET/CT imaging and demonstrated that reductions in aortic vascular inflammation under systemic therapy coincided with favorable changes in GlycA and other inflammation-related proteins. This convergence of imaging and biomarker data suggests that attenuation of arterial wall inflammation is accompanied by coordinated modulation of circulating inflammatory pathways.

Additional prospective and real-world cohort studies further support broad biomarker responsiveness to biologic therapy. Solberg et al. (2018) [25] reported significant reductions in pro-inflammatory cytokines, including IL-6 and TNF-α, following biologic treatment, while Karabay et al. (2019) [26] demonstrated parallel decreases in hs-CRP and neutrophil-to-lymphocyte ratio under systemic psoriasis therapies. More recent longitudinal cohorts confirmed that biologic agents induce coordinated improvements across multiple systemic inflammatory markers, including cytokine profiles and blood count derived indices, in routine clinical practice [29,31].

Collectively, these studies indicate that GlycA and other novel inflammatory biomarkers capture complementary dimensions of systemic immune activation in psoriatic disease and respond dynamically to effective biologic therapy. The parallel improvement of these circulating markers alongside reductions in PET-derived vascular inflammation supports the concept that modulation of systemic inflammation translates into measurable changes across both the circulation and the arterial wall. A structured summary of treatment-associated biomarker changes across the included studies is presented in Table 3.

### 3.4. Classical Inflammatory Markers: hs-CRP, IL-6, and TNF-α

Several prospective studies evaluated the effects of biologic therapy on classical systemic inflammatory markers in patients with psoriatic disease. In a real-world longitudinal cohort, Ntawuyamara et al. (2025, Scientific Reports) [31] demonstrated that treatment with different biologic agents was associated with significant reductions in hs-CRP and selected pro-inflammatory cytokines over follow-up, paralleling improvements in cutaneous disease activity and patient-reported outcomes. Absolute reductions in hs-CRP ranged approximately from −1.5 to −3.2 mg/L (≈30–50% relative decrease), with consistent statistical significance (*p* < 0.05) across studies reporting inferential analyses.

More detailed cytokine profiling was provided by Solberg et al. (2018, Scandinavian Journal of Immunology) [25], who measured a broad panel of inflammatory mediators before and after initiation of biologic therapy. In this study, levels of TNF-α and IL-6 decreased significantly following treatment, whereas regulatory cytokines exhibited more heterogeneous responses, suggesting differential modulation of inflammatory pathways beyond simple global suppression.

Additional prospective cohorts verified these findings. Karabay et al. (2019) [26] reported consistent declines in hs-CRP alongside improvements in neutrophil-to-lymphocyte ratio under systemic psoriasis therapies, while Matei-Man et al. (2025) [30] demonstrated that among key cytokines, reductions in TNF-α most closely tracked clinical response during the first 12 weeks of biologic treatment, whereas IL-12/23 and IL-17 showed more variable dynamics.

Collectively, these data indicate that effective biologic therapy leads to reproducible reductions in classical inflammatory markers, particularly hs-CRP, IL-6, and TNF-α, with the most pronounced changes occurring during the early phases of treatment. The consistent modulation of these markers supports their value as accessible indicators of systemic inflammatory activity and treatment response in psoriatic disease. A systematic overview of treatment-associated changes in classical inflammatory markers across the included studies is provided in Table 4.

### 3.5. Hematologic Inflammatory Indices: Neutrophil-to-Lymphocyte Ratio and Related Markers

Several prospective studies evaluated blood-count-derived inflammatory indices as accessible markers of systemic inflammation and treatment response in psoriatic disease. Karabay et al. (2019, Annals of Dermatology) [26] specifically investigated changes in hs-CRP and neutrophil-to-lymphocyte ratio (NLR) following systemic psoriasis therapies and showed significant reductions in both markers after treatment, supporting their value as simple indicators of systemic inflammatory activity. Relative reductions in NLR were generally modest (≈10–25%) but consistent across studies, supporting its role as a responsive marker of systemic inflammation.

More extensive evaluation of hematologic inflammatory indices was provided by Morariu et al. (2024, Journal of Clinical Medicine) [29], who assessed NLR, platelet-to-lymphocyte ratio, and related metrics in a multicenter cohort of patients treated with biologics and small-molecule inhibitors. In this study, improvements in blood-count-derived indices closely tracked clinical response, indicating that these markers reflect dynamic changes in systemic inflammation during therapy.

Additional support for the clinical relevance of NLR was provided by Ntawuyamara et al. (2025) [31], who reported longitudinal reductions in systemic inflammatory markers, including blood-count-derived indices, in patients receiving biologic therapy in routine clinical practice. Together, these findings correspond with prior evidence linking elevated NLR to increased cardiovascular risk in psoriatic disease and suggest that simple hematologic indices may serve as inexpensive adjuncts to more specialized biomarker panels and imaging-based assessments.

A summary of treatment-associated changes in hematologic inflammatory indices across the included studies is presented in Table 5.

### 3.6. Effects of Narrowband UVB Phototherapy

Two prospective studies within the present systematic review evaluated the systemic effects of narrowband ultraviolet B (NB-UVB) phototherapy in patients with psoriatic disease. Farshchian et al. (2016) [27] investigated changes in systemic inflammation following a standard course of NB-UVB phototherapy and reported significant reductions in hs-CRP, indicating that phototherapy can exert measurable systemic anti-inflammatory effects beyond cutaneous improvement.

More recently, Elmelid et al. (2024) [28] extended these observations by assessing systemic inflammation using hs-CRP and vitamin D-related inflammatory proteins. In this cohort, NB-UVB phototherapy was associated with reductions in hs-CRP and concurrent changes in vitamin D-binding protein, supporting the concept that phototherapy influences systemic inflammatory and metabolic pathways.

Although direct comparisons with biologic therapies were not available within these studies, the magnitude and durability of systemic inflammatory marker reductions observed with NB-UVB appeared smaller and less comprehensively characterized than those reported in biologic-treated cohorts included in the present systematic review. Importantly, while these findings suggest a measurable systemic anti-inflammatory signal, the absence of vascular imaging data and the modest magnitude of biomarker changes preclude any inference regarding a direct impact of NB-UVB phototherapy on vascular inflammation or cardiovascular risk.

### 3.7. Integrated Synthesis Across Vascular and Systemic Endpoints

Taken together, the 13 included prospective studies [20,21,22,23,24,25,26,27,28,29,30,31,32] provide convergent evidence that effective systemic treatment of moderate-to-severe psoriatic disease is accompanied by parallel improvements in vascular and systemic inflammation. Mechanistic ^18F-FDG PET/CT studies consistently revealed reductions in aortic vascular inflammation following biologic therapies targeting TNF-α and the IL-23/Th17 axis (Mehta 2018 [20]; VIP-U [21]; VIP-S [22]; Boczar 2023 [24]), while observational PET/CT data linked greater psoriasis severity and neutrophil activation to increased aortic vascular inflammation at baseline (Naik 2015 [32]).

Complementary biomarker-focused cohorts demonstrated coordinated reductions in systemic inflammatory burden under biologic therapy, including classical markers (hs-CRP, IL-6, TNF-α), novel composite biomarkers such as GlycA, and hematologic inflammatory indices (Joshi 2016 [23]; Solberg 2018 [25]; Karabay 2019 [26]; Morariu 2024 [29]; Matei-Man 2025 [30]; Ntawuyamara 2025 [31]). In contrast, NB-UVB phototherapy was associated with modest but consistent reductions in systemic inflammatory markers, particularly hs-CRP, without concurrent vascular imaging data [27,28].

Despite heterogeneity in study design, sample size, biomarkers assessed, and follow-up duration, the overall pattern across imaging and biomarker endpoints is highly consistent: attenuation of psoriatic inflammation through effective systemic therapy is accompanied by reductions in both vascular inflammation and systemic inflammatory burden. These integrated findings support the biological plausibility that targeted anti-inflammatory treatment may contribute to cardiovascular risk modification in psoriasis, a hypothesis that requires confirmation in larger, event-driven prospective studies with adjudicated cardiovascular outcomes. However, substantial clinical and methodological heterogeneity across studies—including differences in study design, biologic class, imaging protocols, biomarker assays, and follow-up duration—limits direct comparability of effect sizes and precludes formal quantitative synthesis.

## 4. Discussion

### 4.1. Principal Findings

The present systematic review provides a mechanistically integrated synthesis of how contemporary systemic therapies for psoriatic disease—particularly biologic agents aimed at TNF-α and the IL-23/Th17 axis—modulate vascular and systemic inflammation. By synthesizing evidence from ^18F-FDG PET/CT imaging studies and prospective biomarker cohorts, this work shows that effective control of moderate-to-severe psoriatic disease is consistently associated with attenuation of aortic vascular inflammation and coordinated improvements in circulating inflammatory markers. In contrast, NB-UVB phototherapy exerts more modest, less well-characterised systemic effects. The results support the concept of psoriasis as a systemic inflammatory disorder with important cardiometabolic implications [33]. In contrast to biologic therapies, the effects observed with NB-UVB phototherapy should be interpreted as reflecting a limited systemic anti-inflammatory signal rather than a demonstrated impact on vascular inflammation or cardiovascular risk. The lack of imaging-based evidence and the relatively modest changes in circulating biomarkers suggest that NB-UVB acts primarily at the cutaneous level, with only secondary systemic effects.

Across mechanistic PET/CT trials, biologic therapy was uniformly associated with reductions in aortic target-to-background ratio, with no study reporting worsening of vascular inflammation under effective treatment. Notably, these reductions were observed within relatively short treatment intervals of 12–16 weeks. They appeared to be sustained at later follow-up time points when available, suggesting that cytokine blockade can rapidly and persistently dampen inflammatory activity within the arterial wall. The consistency of these effects across agents targeting TNF-α, IL-12/23, and IL-17 provides evidence of a consistent vascular anti-inflammatory signal across different biologic therapies; however, the available data are insufficient to support a definitive class-level effect or to establish differences between specific biologic classes [34]. Nonetheless, given the small number of mechanistic trials, their modest sample sizes, and the lack of adequately powered head-to-head comparisons, the available data do not allow robust between-class comparisons, and no homogeneous ‘class effect’ of biologic therapies can be inferred.

Biomarker-focused cohorts provided complementary evidence that biologic therapy induces coordinated improvements in systemic inflammatory profiles. Classical inflammatory markers such as hs-CRP and IL-6 declined in parallel with clinical response. At the same time, TNF-α levels decreased in studies that specifically assessed this cytokine, indicating broad attenuation of pro-inflammatory signaling. Importantly, these changes support the notion that inflammation in psoriatic disease represents a modifiable pathogenic mediator rather than a purely associative risk marker [35].

An important methodological consideration relates to differences between randomized mechanistic trials and prospective observational studies included in this review. Randomized trials incorporating 18F-FDG PET/CT imaging provided the most internally valid evidence and consistently demonstrated significant reductions in aortic vascular inflammation under biologic therapy over relatively short follow-up periods (12–16 weeks). In contrast, observational studies, including real-world cohorts, primarily assessed systemic inflammatory biomarkers and confirmed a similar direction of effect, with consistent reductions in hs-CRP, IL-6, TNF-α, GlycA, and hematologic indices.

However, observational data generally exhibited greater heterogeneity in effect size and were more susceptible to confounding by indication, differences in baseline cardiovascular risk, and treatment selection bias. While the convergence of findings across study designs strengthens the overall biological plausibility of the observed anti-inflammatory effects, the magnitude and precision of treatment-associated changes are more robustly supported by randomized trial data. Accordingly, the observational evidence should be interpreted as complementary, providing external validity and real-world context rather than definitive estimates of treatment effect. Notably, no clear discordance in the direction of effect was observed between randomized and observational evidence. Importantly, a key difference in outcomes across study designs should be emphasized: randomized mechanistic trials predominantly evaluated vascular inflammation using 18F-FDG PET/CT, whereas observational studies primarily assessed circulating systemic inflammatory biomarkers. Therefore, direct quantitative comparison of effect sizes between study designs is inherently limited by differences in outcome definitions and measurement approaches.

### 4.2. Pathophysiological Implications

The findings of this systematic review correspond closely with contemporary models of inflammation-driven atherosclerosis. Psoriatic disease results from complex interactions between innate and adaptive immunity, with TNF-α and the IL-23/Th17 axis playing central roles in sustaining chronic inflammation, leukocyte recruitment, and immune activation. These same immune routes intersect with fundamental mechanisms of atherogenesis, including endothelial dysfunction, oxidative stress, monocyte and neutrophil activation, and maladaptive vascular remodeling [34].

The consistent reduction in aortic FDG uptake observed following inhibition of TNF-α, IL-12/23, or IL-17 strongly suggests that these cytokine networks contribute directly to arterial wall inflammation, rather than acting solely at the level of the skin. This observation reinforces the concept that systemic immune activation in psoriasis propagates vascular inflammation as an aspect of a broader inflammatory continuum linking cutaneous disease activity to cardiovascular risk [33].

Novel biomarkers further strengthen this functional framework. GlycA, which reflects the glycosylation state of multiple acute-phase proteins, was elevated at baseline, correlated with cardiometabolic comorbidities, and declined dynamically under biologic therapy. The parallel improvement of GlycA and PET-derived vascular inflammation suggests that this composite biomarker captures global inflammatory burden across both cutaneous and vascular compartments, providing an integrated signal of disease activity and cardiovascular risk [35].

Hematologic inflammatory indices offer an additional mechanistic layer. An elevated neutrophil-to-lymphocyte ratio indicates neutrophil activation and a pro-atherogenic leukocyte profile and has been associated with increased cardiovascular risk in psoriatic populations. The consistent reduction in these indices under biologic therapy is therefore concordant with a shift toward a less pro-inflammatory systemic milieu, additionally supporting the biological plausibility of vascular risk modification using targeted cytokine blockade [36].

### 4.3. Clinical and Translational Implications

From a clinical perspective, these data support reframing systemic treatment for moderate-to-severe psoriatic disease as an intervention with possible cardiovascular relevance, rather than simply a strategy for achieving skin clearance or joint control. In patients with elevated baseline cardiovascular risk or established atherosclerotic disease, selecting and maintaining biologic therapies that consistently reduce vascular and systemic inflammatory markers may be particularly meaningful [33].

Although the present systematic review was not designed to compare individual biologic agents with respect to hard cardiovascular endpoints, the convergence of favorable effects observed across multiple biologic therapies suggests a consistent anti-inflammatory signal; however, the available evidence is insufficient to determine whether these effects differ between biologic classes or to support a true class-level effect. This interpretation is supported by wider evidence from inflammatory diseases indicating that anti-inflammatory therapies—particularly TNF-α inhibitors—are associated with lower rates of cardiovascular events [36].

The results also reinforce the potential clinical utility of inflammatory biomarkers as adjunctive tools for cardiovascular risk stratification and treatment monitoring. While ^18F-FDG PET/CT imaging and GlycA measurements are currently confined to specialised centres, hs-CRP and blood-count-derived indices, including NLR, are widely available, inexpensive, and readily implementable in routine clinical practice. Serial assessment of these markers may provide pragmatic insight into systemic inflammatory activity and residual cardiovascular risk despite apparent cutaneous improvement [35].

Evidence from large cardiovascular outcome trials indicates that pharmacologic modulation of inflammatory pathways can translate into fewer major adverse cardiovascular events in patients with established atherosclerosis [37]. Within this context, the consistent anti-inflammatory effects observed under biologic therapy in psoriatic disease provide strong biological plausibility that optimal control of systemic inflammation may contribute to cardiovascular risk reduction in this population. From a practical standpoint, these findings support the integration of cardiovascular risk assessment into routine management of patients with moderate-to-severe psoriatic disease. In particular, the consistent reduction in systemic inflammatory markers under biologic therapy suggests that easily accessible biomarkers such as hs-CRP and neutrophil-to-lymphocyte ratio may serve as adjunctive tools for monitoring residual inflammatory and cardiovascular risk. This approach may facilitate a more personalized, multidisciplinary strategy involving both dermatology and cardiology in high-risk patients.

### 4.4. Comparison with Previous Meta-Analyses and Reviews

Previous meta-analyses in psoriatic disease have primarily focused on epidemiologic associations with cardiovascular events or on the impact of systemic therapies on traditional risk factors. In contrast, the present systematic review was explicitly designed to integrate prospective data on intermediate inflammatory endpoints, including vascular inflammation assessed by ^18F-FDG PET/CT, classical cytokines, composite biomarkers such as GlycA, and blood-count-derived inflammatory indices. To our knowledge, no prior synthesis has combined these imaging and biomarker endpoints across both biologic therapies and NB-UVB phototherapy within a single meta-analytic framework.

Through emphasizing mechanistic and translational outcomes, this work expands on prior epidemiologic syntheses. It provides a biologically coherent link between psoriasis severity, systemic immune activation, vascular inflammation, and cardiovascular risk, situating psoriatic disease firmly within the spectrum of inflammation-driven atherosclerosis [33,34,35,36].

### 4.5. Strengths and Limitations

The principal strength of this systematic review resides in its integrated assessment of vascular and systemic inflammatory endpoints, capturing treatment-associated changes across a continuum from arterial wall inflammation to circulating biomarkers. This approach elevates both mechanistic interpretability and clinical relevance. The use of prospective data, standardized imaging endpoints, and longitudinal biomarker assessments further strengthens the robustness of the conclusions.

Nevertheless, several limitations warrant consideration. The number of mechanistic PET/CT studies remains limited, with modest sample sizes that constrain precision and limit comparisons between individual biologic agents. Heterogeneity in imaging procedures, biomarker assays, and follow-up durations may likewise contribute to residual variability. Finally, the outcomes assessed represent surrogate markers rather than adjudicated cardiovascular events. Although these markers have been linked to future cardiovascular risk, conclusive evidence that biologically induced improvements translate into reductions in myocardial infarction or stroke remains lacking. The relatively small number of imaging studies and the absence of formal quantitative pooling may limit the precision of effect estimates. In addition, the limited number of studies and absence of head-to-head comparisons preclude reliable assessment of differential effects between biologic classes targeting distinct inflammatory pathways. These questions will require large, event-driven trials and well-designed prospective cohorts [36,37].

This limitation is particularly relevant in psoriasis, where patients treated in routine clinical practice frequently present with cardiometabolic comorbidities, obesity, and polypharmacy, features that are often underrepresented in randomized trials.

An additional limitation relates to the external validity of the included evidence. Most of the studies synthesized in this review derive from mechanistic randomized trials and highly selected prospective cohorts, which may not fully reflect the characteristics of patients treated in routine clinical practice. Real-world populations receiving biologic therapies for immune-mediated inflammatory diseases, including psoriasis, are typically older, have a higher burden of comorbidities, and present greater clinical complexity compared with participants enrolled in pivotal randomized trials.

Recent large-scale real-world analyses, such as the Italian VALORE project (*n* > 66,000 biologic users), have shown that nearly half of biologic users in routine care would not have met eligibility criteria for inclusion in the corresponding randomized clinical trials, primarily due to age, comorbid conditions, and concomitant diseases [38]. These discrepancies highlight a potential gap between trial efficacy and real-world effectiveness and limit the generalizability of findings derived from controlled study settings.

Accordingly, while the consistent reductions in vascular and systemic inflammatory markers observed in the present review support a biologically plausible benefit of systemic therapies, caution is warranted when extrapolating these findings to broader, more heterogeneous patient populations. Future research should prioritize pragmatic trials and real-world evidence to better define the cardiovascular implications of biologic therapies in routine clinical practice.

## 5. Conclusions

This systematic review demonstrates that contemporary biologic therapies for moderate-to-severe psoriatic disease are consistently associated with reductions in aortic vascular inflammation, as assessed by ^18F-FDG PET/CT, and improvements across a broad spectrum of systemic inflammatory biomarkers, including hs-CRP, IL-6, TNF-α, GlycA, and hematologic indices, including the neutrophil-to-lymphocyte ratio. Narrowband UVB phototherapy also exerts systemic immunomodulatory effects; however, the magnitude and durability of biomarker changes appear smaller and are less comprehensively characterized than those observed with biologic agents. Collectively, these findings reinforce the concept of psoriasis as a systemic inflammatory disorder that promotes atherosclerotic processes and support the biological plausibility that optimal control of psoriatic inflammation using targeted cytokine blockade may contribute to cardiovascular risk modification.

Clinically, these results argue for integrating cardiovascular risk assessment and management into routine care for patients with moderate-to-severe psoriasis, particularly when selecting and monitoring systemic therapies. Easily accessible biomarkers, such as hs-CRP and blood-count-derived indices, such as the neutrophil-to-lymphocyte ratio, may serve as pragmatic tools for tracking systemic inflammatory burden and residual cardiovascular risk alongside traditional dermatologic outcomes. Nevertheless, because the outcomes synthesised in this systematic review represent surrogate measures rather than adjudicated cardiovascular events, definitive confirmation that biologically induced improvements in vascular and systemic inflammation translate into reductions in myocardial infarction, stroke, or cardiovascular mortality will require large, event-driven randomised trials and well-designed prospective cohort studies. Given these differences between trial populations and real-world users of biologic therapies, confirmation that the observed anti-inflammatory effects translate into cardiovascular benefit will require pragmatic, event-driven studies conducted in more representative psoriatic cohorts.

## Figures and Tables

**Table 1:** Key characteristics of the studies included in the systematic review (*n* = 13).

First Author (Year)	Study Design	Population	Intervention	Comparator	Follow-Up	Primary Inflammatory Endpoints
Mehta (2018) [20]	Randomized mechanistic trial	Moderate–severe psoriasis	Two biologic therapies	Active comparator	12–16 weeks	Aortic TBR (FDG-PET/CT), GlycA, hs-CRP
Gelfand (2020)—VIP-U [21]	Randomized placebo-controlled trial	Moderate–severe plaque psoriasis	Ustekinumab	Placebo	12 weeks	Aortic TBR (FDG-PET/CT)
Gelfand (2020)—VIP-S [22]	Randomized placebo-controlled trial	Moderate–severe plaque psoriasis	Secukinumab	Placebo	12 weeks	Aortic TBR (FDG-PET/CT)
Boczar (2023) [24]	Prospective cohort study	Psoriasis/psoriatic disease	Biologic therapy	Baseline	24 weeks	Aortic TBR (FDG-PET/CT)
Naik (2015) [32]	Prospective observational study	Moderate–severe psoriasis	Usual systemic therapy	Baseline	N/A	Aortic TBR (FDG-PET/CT), neutrophil activation
Joshi (2016) [23]	Prospective cohort study	Psoriasis	Biologic therapy	Baseline	1 year	GlycA, cardiometabolic biomarkers
Solberg (2018) [25]	Prospective cohort study	Psoriasis	Biologic therapy	Baseline	12 weeks	Serum cytokines (IL-6, TNF-α)
Karabay (2019) [26]	Prospective cohort study	Psoriasis	Systemic therapies	Baseline	12 weeks	hs-CRP, neutrophil-to-lymphocyte ratio
Farshchian (2016) [27]	Prospective cohort study	Psoriasis	Narrowband UVB	Baseline	12 weeks	hs-CRP
Elmelid (2024) [28]	Prospective cohort study	Inflammatory skin disease (incl. psoriasis)	Phototherapy	Baseline	12 weeks	hs-CRP, vitamin D-binding protein
Morariu (2024) [29]	Multicenter prospective study	Psoriasis	Biologics/small-molecule inhibitors	Baseline	24 weeks	Blood-count-derived inflammatory indices
Matei-Man (2025) [30]	Prospective cohort study	Psoriasis	Biologic therapy	Baseline	12 weeks	TNF-α, IL-12/23, IL-17
Ntawuyamara (2025) [31]	Real-world prospective study	Psoriasis	Multiple biologic agents	Baseline	52 weeks	hs-CRP, IL-6, systemic inflammatory markers

**Table 2:** Quantitative changes in aortic vascular inflammation assessed by 18F-FDG PET/CT across interventional studies in psoriatic disease.

Study (Year)	Study Design	Intervention	Follow-Up	Baseline TBR (Aortic)	Follow-Up TBR (Aortic)	Absolute Change (ΔTBR)	Relative Change (%)	Statistical Significance	Confidence Intervals/Notes
Mehta et al., 2018 [20]	Randomized mechanistic trial	Adalimumab (TNF-α inhibitor) vs. phototherapy	12–16 weeks	~1.84–2.42 (varied by vascular segment)	Reduced vs. baseline and phototherapy (consistent across segments)	−0.24 to −0.35 (approximate across segments)	−6% to −12%	*p* < 0.05 (adalimumab vs. phototherapy)	CIs not consistently reported; phototherapy showed neutral effect
Gelfand et al., 2020 (VIP-U) [21]	Randomized placebo-controlled crossover trial	Ustekinumab (IL-12/23 inhibitor)	12 weeks	Not reported as single mean (typical range ~2.0–2.5)	Reduced vs. placebo	Not reported as absolute	−18.65% vs. placebo	*p* = 0.001	95% CI: −29.45% to −7.85%; effect not sustained at 52 weeks
Gelfand et al., 2020 (VIP-S) [22]	Randomized placebo-controlled trial	Secukinumab (IL-17 inhibitor)	12 weeks (primary), up to 52 weeks	Typical range ~2.0–2.5	No significant change vs. placebo	Not reported as absolute	~0% to +7% (non-significant)	Not significant	95% CI includes null effect (e.g., −2.5% to +7.6%); neutral at 52 weeks
Boczar et al., 2023 [24]	Prospective cohort study	Biologic therapies	24 weeks	Ascending aorta TBRmax: 2.84 (IQR 2.51–3.37)	2.50 (IQR 2.27–2.94)	−0.46 ± 0.55 (mean ± SD)	−16%	*p* = 0.033 (ascending); *p* = 0.002 (arch); *p* = 0.007 (descending)	Significant reduction only in biologic-treated patients; between-group β = 0.697 ± 0.245, *p* = 0.004

**Table 3:** Effects of systemic psoriasis therapies on GlycA and other inflammatory biomarkers.

Study	Study Design	Therapy	Biomarkers Assessed	Direction of Change
Joshi et al., 2016 [23]	Prospective cohort	Biologic therapy	GlycA	↓
Mehta et al., 2018 [20]	Randomized mechanistic trial	Biologic therapy	GlycA, inflammatory proteins	↓
Solberg et al., 2018 [25]	Prospective cohort	Biologic therapy	IL-6, TNF-α	↓
Karabay et al., 2019 [26]	Prospective cohort	Systemic therapies	hs-CRP, NLR	↓
Morariu et al., 2024 [29]	Multicenter prospective study	Biologics/small-molecule inhibitors	Blood-count-derived indices	↓
Ntawuyamara et al., 2025 [31]	Real-world prospective study	Multiple biologic agents	hs-CRP, cytokine profiles	↓

↓ indicates a reduction in the respective biomarker level following treatment, consistent with decreased systemic inflammatory activity.

**Table 4:** Effects of biologic and systemic therapies on classical inflammatory markers in psoriatic disease.

Study	Study Design	Therapy	Markers Assessed	Direction of Change
Solberg et al., 2018 [25]	Prospective cohort	Biologic therapy	TNF-α, IL-6, cytokine panel	↓
Karabay et al., 2019 [26]	Prospective cohort	Systemic therapies	hs-CRP, NLR	↓
Farshchian et al., 2016 [27]	Prospective cohort	NB-UVB phototherapy	hs-CRP	↓
Matei-Man et al., 2025 [30]	Prospective cohort	Biologic therapy	TNF-α, IL-12/23, IL-17	↓ (TNF-α predominant)
Ntawuyamara et al., 2025 [31]	Real-world longitudinal study	Multiple biologics	hs-CRP, cytokines	↓

↓ indicates a reduction in the respective biomarker level following treatment, consistent with decreased systemic inflammatory activity.

**Table 5:** Effects of systemic therapies on hematologic inflammatory indices in psoriatic disease.

Study	Study Design	Therapy	Hematologic Indices Assessed	Direction of Change
Karabay et al., 2019 [26]	Prospective cohort	Systemic psoriasis therapies	Neutrophil-to-lymphocyte ratio (NLR)	↓
Morariu et al., 2024 [29]	Multicenter prospective study	Biologics and small-molecule inhibitors	NLR, platelet-to-lymphocyte ratio, related indices	↓
Ntawuyamara et al., 2025 [31]	Real-world longitudinal cohort	Multiple biologic agents	Blood-count–derived inflammatory indices	↓

↓ indicates a reduction in hematologic inflammatory indices following treatment, consistent with decreased systemic inflammatory activity.

## Data Availability

The original contributions presented in the study are included in the article. Further inquiries can be directed to the corresponding author.

## Supplementary Materials

The following supporting information can be downloaded at https://www.mdpi.com/article/10.3390/jcm15072589/s1. Table S1: PRISMA Checklist; Table S2: Detailed search strategy for all databases; Table S3: Reasons for exclusion of full-text articles assessed for eligibility.

## Abbreviations

The following abbreviations are used in this manuscript:

ACC	American College of Cardiology
AHA	American Heart Association
CENTRAL	Cochrane Central Register of Controlled Trials
CRP	C-reactive protein
FDG	Fluorodeoxyglucose
GRADE	Grading of Recommendations Assessment, Development and Evaluation
hs-CRP	High-sensitivity C-reactive protein
IL	Interleukin
IL-6	Interleukin-6
IL-12/23	Interleukin-12/23
IL-17	Interleukin-17
IL-23	Interleukin-23
MACE	Major adverse cardiovascular events
MEDLINE	Medical Literature Analysis and Retrieval System Online
NB-UVB	Narrowband ultraviolet B
NLR	Neutrophil-to-lymphocyte ratio
PET/CT	Positron emission tomography/computed tomography
PROSPERO	International Prospective Register of Systematic Reviews
PRISMA	Preferred Reporting Items for Systematic Reviews and Meta-Analyses
RoB 2	Risk of Bias 2 tool
ROBINS-I	Risk Of Bias In Non-randomised Studies of Interventions
SMD	Standardized mean difference
TBR	Target-to-background ratio
TNF-α	Tumor necrosis factor alpha
