# Supplementary material for: Effects of Biologic Therapies and Narrowband UVB Phototherapy on Vascular Inflammation and Systemic Inflammatory Biomarkers in Psoriasis: A Systematic Review and Narrative Synthesis of Prospective Studies
Source: J Clin Med. 2026 Mar 28;15(7):2589. doi: 10.3390/jcm15072589 (PMC13073588; doi:10.3390/jcm15072589)
Supplement: Supplementary file 1 [file jcm-15-02589-s001.zip › jcm-4199015-supplementary.pdf]

Table S1. PRISMA Checklist.

Section and Topic	Item #	Checklist item	Location where item is reported
**TITLE**			
Title	1	Identify the report as a systematic review.	Location: Title page (“Systematic Review” and title)
**ABSTRACT**			
Abstract	2	See the PRISMA 2020 for Abstracts checklist.	Location: Abstract
**INTRODUCTION**			
Rationale	3	Describe the rationale for the review in the context of existing knowledge.	Location: Introduction, paragraphs 1–5
Objectives	4	Provide an explicit statement of the objective(s) or question(s) the review addresses.	Location: End of Introduction (final paragraph)
**METHODS**			
Eligibility criteria	5	Specify the inclusion and exclusion criteria for the review and how studies were grouped for the syntheses.	Location: Section 2.2 Eligibility Criteria
Information sources	6	Specify all databases, registers, websites, organisations, reference lists and other sources searched or consulted to identify studies. Specify the date when each source was last searched or consulted.	Location: Section 2.3 Information Sources and Search Strategy
Search strategy	7	Present the full search strategies for all databases, registers and websites, including any filters and limits used.	Location: Section 2.3 and Supplementary Table S2
Selection process	8	Specify the methods used to decide whether a study met the inclusion criteria of the review, including how many reviewers screened each record and each report retrieved, whether they worked independently, and if applicable, details of automation tools used in the process.	Location: Section 2.4 Study Selection
Data collection process	9	Specify the methods used to collect data from reports, including how many reviewers collected data from each report, whether they worked independently, any processes for obtaining or confirming data from study investigators, and if applicable, details of automation tools used in the process.	Location: Section 2.5 Data Collection Process
Data items	10a	List and define all outcomes for which data were sought. Specify whether all results that were compatible with each outcome domain in each study were sought (e.g. for all measures, time points, analyses), and if not, the methods used to decide which results to collect.	Location: Section 2.6 Data Items and Outcome Definitions
	10b	List and define all other variables for which data were sought (e.g. participant and intervention characteristics, funding sources). Describe any assumptions made about any missing or unclear information.	Location: Section 2.5 Data Collection Process
Study risk of bias assessment	11	Specify the methods used to assess risk of bias in the included studies, including details of the tool(s) used, how many reviewers assessed each study and whether they worked independently, and if applicable, details of automation tools used in the process.	Location: Section 2.7 Risk of Bias Assessment
Effect measures	12	Specify for each outcome the effect measure(s) (e.g. risk ratio, mean difference) used in the synthesis or presentation of results.	Location: Section 2.8 Effect Measures
Synthesis	13a	Describe the processes used to decide which studies were eligible for each synthesis (e.g. tabulating the study intervention	Location: Section 2.9 Synthesis

Section and Topic	Item #	Checklist item	Location where item is reported
methods		characteristics and comparing against the planned groups for each synthesis (item #5)).	Methods
	13b	Describe any methods required to prepare the data for presentation or synthesis, such as handling of missing summary statistics, or data conversions.	Location: Section 2.8 Effect Measures
	13c	Describe any methods used to tabulate or visually display results of individual studies and syntheses.	Location: Section 2.9 Synthesis Methods
	13d	Describe any methods used to synthesize results and provide a rationale for the choice(s). If meta-analysis was performed, describe the model(s), method(s) to identify the presence and extent of statistical heterogeneity, and software package(s) used.	Location: Section 2.9 Synthesis Methods
	13e	Describe any methods used to explore possible causes of heterogeneity among study results (e.g. subgroup analysis, meta-regression).	Location: Section 2.10 Subgroup and Sensitivity Analyses
	13f	Describe any sensitivity analyses conducted to assess robustness of the synthesized results.	Location: Section 2.10 Subgroup and Sensitivity Analyses
Reporting bias assessment	14	Describe any methods used to assess risk of bias due to missing results in a synthesis (arising from reporting biases).	Location: Section 2.11 Reporting Bias and Certainty of Evidence
Certainty assessment	15	Describe any methods used to assess certainty (or confidence) in the body of evidence for an outcome.	Location: Section 2.11 Reporting Bias and Certainty of Evidence
**RESULTS**			
Study selection	16a	Describe the results of the search and selection process, from the number of records identified in the search to the number of studies included in the review, ideally using a flow diagram.	Location: Section 3.1 Study Selection and Figure 1 (PRISMA diagram)
	16b	Cite studies that might appear to meet the inclusion criteria, but which were excluded, and explain why they were excluded.	Location: Section 2.4 Study Selection and Supplementary Table S3
Study characteristics	17	Cite each included study and present its characteristics.	Location: Section 3.1 and Table 1
Risk of bias in studies	18	Present assessments of risk of bias for each included study.	Section 2.7 Risk of Bias Assessment and Results Sections 3.2–3.7 (risk of bias incorporated into the narrative synthesis)
Results of individual studies	19	For all outcomes, present, for each study: (a) summary statistics for each group (where appropriate) and (b) an effect estimate and its precision (e.g. confidence/credible interval), ideally using structured tables or plots.	Location: Tables 1–4 and Sections 3.2–3.6
Results of syntheses	20a	For each synthesis, briefly summarise the characteristics and risk of bias among contributing studies.	Location: Sections 3.2–3.7
	20b	Present results of all statistical syntheses conducted. If meta-analysis was done, present for each the summary estimate and its precision (e.g. confidence/credible interval) and measures of	Location: Sections 3.2–3.7

Section and Topic	Item #	Checklist item	Location where item is reported
		statistical heterogeneity. If comparing groups, describe the direction of the effect.	
	20c	Present results of all investigations of possible causes of heterogeneity among study results.	Section 2.10 Subgroup and Sensitivity Analyses (no quantitative subgroup analyses were feasible due to heterogeneity)
	20d	Present results of all sensitivity analyses conducted to assess the robustness of the synthesized results.	Section 2.10 Subgroup and Sensitivity Analyses (no quantitative subgroup analyses were feasible due to heterogeneity)
Reporting biases	21	Present assessments of risk of bias due to missing results (arising from reporting biases) for each synthesis assessed.	Section 2.11 Reporting Bias and Certainty of Evidence (qualitative assessment)
Certainty of evidence	22	Present assessments of certainty (or confidence) in the body of evidence for each outcome assessed.	Location: Section 2.11 Reporting Bias and Certainty of Evidence
**DISCUSSION**			
Discussion	23a	Provide a general interpretation of the results in the context of other evidence.	Location: Section 4.1 Principal Findings
	23b	Discuss any limitations of the evidence included in the review.	Location: Section 4.5 Strengths and Limitations
	23c	Discuss any limitations of the review processes used.	Location: Section 4.5 Strengths and Limitations
	23d	Discuss implications of the results for practice, policy, and future research.	Location: Sections 4.3 and 5 Conclusions
**OTHER INFORMATION**			
Registration and protocol	24a	Provide registration information for the review, including register name and registration number, or state that the review was not registered.	Location: Section 2.1 Protocol and Reporting (PROSPERO CRD420261296646)
	24b	Indicate where the review protocol can be accessed, or state that a protocol was not prepared.	Location: Section 2.1 Protocol and Reporting
	24c	Describe and explain any amendments to information provided at registration or in the protocol.	Location: Not applicable (no protocol amendments reported)
Support	25	Describe sources of financial or non-financial support for the	Location: Funding

Section and Topic	Item #	Checklist item	Location where item is reported
		review, and the role of the funders or sponsors in the review.	section
Competing interests	26	Declare any competing interests of review authors.	Location: Conflicts of Interest section
Availability of data, code and other materials	27	Report which of the following are publicly available and where they can be found: template data collection forms; data extracted from included studies; data used for all analyses; analytic code; any other materials used in the review.	Data Availability Statement and Supplementary Materials

Table S2. Detailed search strategy for all databases.

Database	Search strategy
MEDLINE (via PubMed)	(psoriasis[MeSH Terms] OR psoriasis[Title/Abstract] OR psoriatic arthritis[Title/Abstract]) AND (biologic therapy[Title/Abstract] OR biologics[Title/Abstract] OR tumor necrosis factor inhibitor*[Title/Abstract] OR TNF inhibitor*[Title/Abstract] OR interleukin 17 inhibitor*[Title/Abstract] OR interleukin 23 inhibitor*[Title/Abstract] OR IL-17[Title/Abstract] OR IL-23[Title/Abstract] OR ustekinumab[Title/Abstract] OR secukinumab[Title/Abstract] OR ixekizumab[Title/Abstract] OR guselkumab[Title/Abstract] OR risankizumab[Title/Abstract] OR tildrakizumab[Title/Abstract] OR narrowband UVB[Title/Abstract] OR NB-UVB[Title/Abstract] OR phototherapy[Title/Abstract]) AND (vascular inflammation[Title/Abstract] OR FDG PET[Title/Abstract] OR PET CT[Title/Abstract] OR positron emission tomography[Title/Abstract] OR target to background ratio[Title/Abstract] OR TBR[Title/Abstract] OR C-reactive protein[Title/Abstract] OR hs-CRP[Title/Abstract] OR interleukin-6[Title/Abstract] OR IL-6[Title/Abstract] OR TNF-alpha[Title/Abstract] OR GlycA[Title/Abstract] OR neutrophil-to-lymphocyte ratio[Title/Abstract] OR NLR[Title/Abstract])
Embase	('psoriasis'/exp OR psoriasis:ti,ab OR 'psoriatic arthritis':ti,ab) AND ('biologic therapy'/exp OR biologic*:ti,ab OR 'tumor necrosis factor inhibitor':ti,ab OR 'interleukin 17 inhibitor':ti,ab OR 'interleukin 23 inhibitor':ti,ab OR ustekinumab:ti,ab OR secukinumab:ti,ab OR ixekizumab:ti,ab OR guselkumab:ti,ab OR risankizumab:ti,ab OR tildrakizumab:ti,ab OR 'narrowband uvb':ti,ab OR phototherapy:ti,ab) AND ('vascular inflammation':ti,ab OR 'fdg pet':ti,ab OR 'positron emission tomography'/exp OR 'target to background ratio':ti,ab OR 'c reactive protein':ti,ab OR 'interleukin 6':ti,ab OR 'tumor necrosis factor alpha':ti,ab OR glycA:ti,ab OR 'neutrophil lymphocyte ratio':ti,ab)
Web of Science Core Collection	TS=(psoriasis OR "psoriatic arthritis") AND TS=(biologic* OR "tumor necrosis factor inhibitor*" OR "interleukin 17 inhibitor*" OR "interleukin 23 inhibitor*" OR ustekinumab OR secukinumab OR ixekizumab OR guselkumab OR risankizumab OR tildrakizumab OR "narrowband UVB" OR phototherapy) AND TS=("vascular inflammation" OR "FDG PET" OR "PET CT" OR "target to background ratio" OR TBR OR "C-reactive protein" OR

Database	Search strategy
	hs-CRP OR "interleukin-6" OR IL-6 OR "TNF-alpha" OR GlycA OR "neutrophil-to-lymphocyte ratio" OR NLR)
Scopus	TITLE-ABS-KEY (psoriasis OR "psoriatic arthritis") AND TITLE-ABS-KEY (biologic* OR "tumor necrosis factor inhibitor*" OR "interleukin 17 inhibitor*" OR "interleukin 23 inhibitor*" OR ustekinumab OR secukinumab OR ixekizumab OR guselkumab OR risankizumab OR tildrakizumab OR "narrowband UVB" OR phototherapy) AND TITLE-ABS-KEY ("vascular inflammation" OR "FDG PET" OR "PET CT" OR "target to background ratio" OR TBR OR "C-reactive protein" OR hs-CRP OR "interleukin-6" OR IL-6 OR "TNF-alpha" OR GlycA OR "neutrophil-to-lymphocyte ratio" OR NLR)
Cochrane CENTRAL	(psoriasis OR psoriatic arthritis) AND (biologic therapy OR TNF inhibitor OR IL-17 inhibitor OR IL-23 inhibitor OR ustekinumab OR secukinumab OR ixekizumab OR guselkumab OR risankizumab OR tildrakizumab OR narrowband UVB OR phototherapy) AND (vascular inflammation OR FDG PET OR PET CT OR C-reactive protein OR interleukin-6 OR TNF-alpha OR GlycA OR neutrophil-to-lymphocyte ratio)

Table S3. Reasons for exclusion of full-text articles assessed for eligibility.

**Reason for exclusion**	**Number of studies (n)**	**Examples of excluded studies**
Cross-sectional design without longitudinal follow-up	48	Studies assessing vascular inflammation or biomarkers at a single time point without post-treatment evaluation
Retrospective study design	36	Retrospective database or chart review studies lacking prospective follow-up
No relevant outcomes (no PET/CT or systemic inflammatory biomarkers reported longitudinally)	42	Studies reporting only clinical dermatologic outcomes without inflammatory markers
Population not meeting eligibility criteria	18	Studies including heterogeneous inflammatory conditions without separate psoriasis analysis
Intervention not eligible	14	Studies evaluating topical therapies or non-eligible interventions
Conference abstract or insufficient data	11	Abstract-only publications without extractable quantitative data
Case reports or small case series (<10 participants)	8	Case reports or very small cohorts
Duplicate or overlapping cohort	7	Secondary analyses or duplicate publications

Reason for exclusion	Number of studies (n)	Examples of excluded studies
Non-human or mechanistic laboratory study	5	Animal or in vitro studies
Full text not available	2	Articles without accessible full text

Total excluded = 191
